# Genetic differences between suicide deaths and deaths of undetermined intent

**DOI:** 10.1111/sltb.12926

**Published:** 2022-10-31

**Authors:** Alexis C. Edwards, Henrik Ohlsson, Eve K. Mościcki, Jan Sundquist, Casey Crump, Kenneth S. Kendler, Kristina Sundquist

**Affiliations:** ^1^ Department of Psychiatry, Virginia Institute for Psychiatric and Behavioral Genetics Virginia Commonwealth University Richmond Virginia USA; ^2^ Center for Primary Health Care Research Lund University Malmö Sweden; ^3^ Department of Family Medicine and Community Health Icahn School of Medicine at Mount Sinai New York New York USA

**Keywords:** aggregate genetics, psychiatric comorbidity, suicide, undetermined intent death

## Abstract

**Introduction:**

Few, if any, prior studies have considered whether undetermined intent (UDI) deaths and suicide deaths differ with respect to genetic liability for suicidal behavior or psychopathology.

**Methods:**

The authors used Swedish national registry data to identify suicide deaths (*N* = 31,835) and UDI deaths (*N* = 10,623); sociodemographic covariates; and registrations for psychopathology. Family genetic risk scores (FGRS) were derived for each form of psychopathology. The authors used LASSO models to assess genetic and phenotypic differences across outcomes.

**Results:**

In the multivariate LASSO regressions, higher FGRS for major depression, bipolar disorder, and suicide death were associated with lower odds of UDI relative to unambiguous suicide (OR = 0.91–0.95), while those for alcohol and drug use disorders, ADHD, and criminal behavior were associated with higher odds of UDI relative to unambiguous suicide (OR = 1.04–1.12). When the corresponding phenotypic registration status for these outcomes was included in a subsequent model, the associations were attenuated and of small magnitude, but many remained different from OR = 1.

**Conclusions:**

Aggregate genetic differences between unambiguous suicide decedents and UDI deaths are small, particularly when accounting for psychiatric comorbidity, but in some cases, statistically significant. These findings suggest that different analytic treatment of UDI deaths may be warranted depending on the research question. Replication in other samples, and using molecular genetic data, is necessary.

## INTRODUCTION

Suicide is a persistent public health concern, with approximately 7,00,000 annual deaths per year worldwide (World Health Organization, 2021). Risk for suicide is complex and multifaceted. In addition to sociodemographic factors (e.g., age, sex, race/ethnicity [World Health Organization, 2021]) and environmental exposures (e.g., divorce, unemployment [Fjeldsted et al., 2017; Nordt et al., 2015]), suicide risk is also attributable to genetic liability, with twin/family studies reporting heritability estimates of ~0.3–0.7 across suicidal outcomes (Althoff et al., 2012; Edwards, Ohlsson, Mościcki, et al., 2021; Pedersen & Fiske, 2010).

The classification of suicide deaths is a topic of ongoing discussion within the field of suicide research. Specifically, whether deaths of “undetermined intent” (UDI) should be included as suicide deaths or considered separately is of concern. Certification of deaths under “circumstances undetermined” was introduced with the eighth revision if the International Classification of Diseases (ICD‐8). Prior to this, in ICD‐7, deaths from self‐inflicted injury were classified as suicides by default unless there was a specific statement that the injury was unintentional. In the US, the change in classification from ICD‐7 to ICD‐8 accounted for an approximate decrease in recognized suicide deaths of 5%–6% after what was considered an artifactual increase in suicide by 3% (O'Carroll, 1989). Such shifts introduce uncertainty into our understanding of how widespread suicide is and could inappropriately result in the reallocation of resources for prevention.

Recent studies have sought to determine the extent to which UDI deaths lead to an undercount of suicides, with inconsistent results. Various reviews of death records and supplemental information (e.g., medical records, police interviews) have suggested that nontrivial proportions of UDI deaths are true suicides, and that appropriate reclassification would increase the number of suicide deaths by ~5%–28% (Allebeck et al., 1991; Bakst et al., 2016; Chang et al., 2010; Connolly & Cullen, 1995). Others have considered whether characteristics – such as age, sex, or psychiatric history – of individuals whose deaths are classified as UDI versus suicide deaths differ. Bjorkenstam et al. (2014), examining deaths in Sweden between 1987–2011, found that sociodemographic features such as sex, marital status, and educational attainment differed across groups; furthermore, those whose deaths were classified as suicides rather than UDI were more likely to have a history of psychiatric hospitalization, though no differences were observed across groups with respect to neuroleptics, sedatives, or antidepressants prescription use. Sex and marital status, but not educational status, also differed in a study of Canadian deaths (Lachaud et al., 2018). While the method of death contributes to a medical examiner's ability to determine intent, Chang et al. (2010) found that individuals who died by suffocation and poisoning had similar age, sex, and marital status profiles regardless of whether their deaths were classified as suicides or of UDI. Thus, while some differences across groups have been observed, it seems likely that UDI deaths include a nontrivial number of suicides.

Given the heritable nature of suicidal behavior, it is feasible that these groups may differ with respect to underlying genetic liability. Prior research using traditional biometric models, a family history‐based estimation of aggregate genetic liability, or molecular genetic data indicates that suicidal behavior is genetically related to mood and substance use disorders and other forms of psychopathology (Docherty et al., 2020; Edwards, Ohlsson, Lannoy, et al., 2021; Kendler, Ohlsson, Mościcki, et al., 2021; Mullins et al., 2022). In the current study, we expand the question of genetic influences on suicidal behavior to UDI deaths, asking whether we observe differences in genetic liability to various psychiatric/behavioral outcomes across individuals whose deaths were classified as UDI versus unambiguous suicides. We benefit from longitudinal Swedish national registries, which we use to derive family genetic risk scores (FGRS) for major psychiatric disorders, substance use disorders, criminal behavior, and suicidal behavior. FGRS were recently developed to capture familial risk for a specified phenotype/trait in the absence of individual‐level molecular genetic data, adjusting for a shared family environment. Previous studies have employed FGRS to examine whether individual risk for a particular outcome is related to genetic liability for a range of distinct phenotypes (Kendler, Ohlsson, Sundquist, & Sundquist, 2021a, 2021b), revealing, for example, genetic distinctions between affective and psychotic disorders (Kendler, Ohlsson, Mościcki, et al., 2021; Kendler, Ohlsson, Sundquist, & Sundquist, 2021a, 2021b). By using FGRS to assess whether suicide versus UDI decedents exhibit pronounced genetic differences, we hope to gain insight to potential etiologic differences and inform decisions about whether to include UDI deaths alongside unambiguous suicide deaths in suicide research.

## MATERIALS AND METHODS

### Sample

We collected longitudinal information on individuals from Swedish population‐based registers with national coverage linking each person's unique personal identification number, which, to preserve confidentiality, was replaced with a serial number by Statistics Sweden. We secured ethical approval for this study from the Regional Ethical Review Board in Lund, and no participant consent was required as the analyses were based on secondary data (No. 2008/409 and later amendments).

### Outcome

In the database, we included all individuals aged 15 and older registered for deaths from suicide and UDI in the Swedish mortality register. The register covers the period from 1969 to 2018. Furthermore, we required that the individual was born in Sweden between 1932 and 1995 to Swedish born parents. Suicide was defined using ICD codes X60–X84 and Y10–Y34 (ICD10) and E950–E959 (ICD8/9); UDI was defined using Y10–Y34 (ICD10) and E980–E989 (ICD8/9).

### Covariates and predictors

We included year of birth, sex, parental education, county of residence at the date of suicide, and a set of Family Genetic Risk Scores. We included county of residence to account for potential regional differences in practice regarding the classification of deaths as suicide versus UDI. The individual FGRS included Major Depression (MD), Anxiety Disorders (AD), Obsessive–Compulsive Disorder (OCD), Bipolar Disorder (BD), Schizophrenia (SZ), Bulimia (BUL), Anorexia Nervosa (AN), Alcohol Use Disorder (AUD), Drug Use Disorder (DUD), Criminal Behavior (CB), Attention Deficit Hyperactivity Disorder (ADHD), Suicide Death (SD), and Suicide Attempt (SA). The FGRS were based on selected 1st, 2nd, 3rd, 4th, and 5th degree relatives to the probands, with a mean of 40.1 relatives per proband. In the database, we also included the date of first registration for the 12 of the 13 phenotypes above (excluding SD, since SD is one level of the outcome) used for FGRS calculation. Briefly, FGRS leverage information on the presence or absence, across a range of national registries, of an outcome of interest in a proband's 1st to 5th degree relatives. We account for the proband's genetic relatedness to each relative (e.g., 0.5 for parents and full siblings, 0.125 for first cousins), the relative's age at first registrations for the outcome (where applicable), the proband's years of cohabitation with each relative, population prevalence of the outcome (reflecting rates adjusted for sex and year of birth), and other relevant factors. Complete details on FGRS derivation and definition of variables are provided in the Tables S1 and S2. Thus, the scores ultimately reflect an individual's genetic liability for a particular outcome.

### Statistical analyses

We used logistic regression to estimate associations between FGRS and unambiguous suicides (coded as 0) versus UDI deaths (coded as 1). First, we conducted separate models for each of the FGRS while controlling for year of birth, sex, county of residence, and parental education. To identify the most parsimonious model, we used Least Absolute Shrinkage and Selection Operator (LASSO) regression for variable selection among the FGRS. The goal of LASSO regression is to obtain the subset of predictors that minimizes prediction error for the response variable. We used the HPGENSELECT (Lund, 2017) procedure in SAS with LASSORHO = 0.80 and LASSOSTEPS = 20 to select the model with the smallest SBC. The Schwarz criterion (SBC) is a criterion for model selection among a finite set of models, and it is based, in part, on the likelihood function. After the selection of FGRS, we included the year of birth, sex, county of residence, and parental education in the logistic regression model. In the final model, we also included phenotypic registrations that corresponded to the selected FGRS (e.g., for major depression, alcohol use disorder, etc.). As described in detail in Table S1, these registrations are derived from registries for primary care, outpatient care, hospitals, prescriptions, and criminality. All the analyses were performed using SAS 9.4 (©2002–2012 SAS Institute Inc., Cary NC US). We used guidelines suggested by Chen et al. (2010) to interpret effect sizes, with OR = 1.68 as small, OR = 3.47 as a medium, and OR = 6.71 as large.

## RESULTS

### Descriptive statistics and univariate analyses

There was a total of *N* = 42,548 suicide deaths and deaths of undetermined intent (28.35% female) in the cohort born 1932–1995 to Swedish‐born parents (Table 1). Of these, 25.02% were deaths of undetermined intent. The mean age at death was in the early 40 s for both unambiguous suicide deaths (42.9 [*SD* = 14.7]) and deaths of undetermined intent (42.7 [*SD* = 13.6]), with no significant difference across groups (*t* = 1.29, *p* = 0.20). The standardized mean parental education was lower among the UDI decedents than among unambiguous suicide decedents (*t* = 6.06, *p* < 0.0001).

**TABLE 1:** Sample characteristics^a^

	Total	Female	Male
Total deaths (*N*)	42,458	12,037	30,421
15–24	4636	1268	3368
25–34	8096	2105	5991
35–44	9976	2707	7269
45–54	9518	2836	6682
55–64	6315	1963	4352
65–74	2998	901	2097
75 and older	919	257	662
*N* (%) Undetermined intent deaths	10,623 (25.02%)	3110 (25.84%)	7513 (24.70%)
*N* (%) of Deaths classified as UDI, by age at death			
15–24	1028 (22.2%)	257 (20.3%)	771 (22.9%)
25–34	1971 (24.4%)	482 (22.9%)	1489 (24.9%)
35–44	2721 (27.3%)	698 (25.8%)	2.023 (27.8%)
45–54	2528 (26.6%)	786 (27.7%)	1742 (26.1%)
55–64	1545 (24.5%)	572 (29.1%)	973 (22.4%)
65–74	688 (23.0%)	256 (28.4%)	432 (20.6%)
75 and older	142 (15.5%)	59 (23.0%)	83 (12.5%)
*N* (%) of Deaths classified as UDI, by parental education^b^			
Below −1 *SD*	438 (27.0%)	119 (28.5%)	319 (26.5%)
−1 to 0 *SD*	4950 (25.3%)	1392 (26.6%)	3558 (24.8%)
0 to 1 *SD*	4440 (25.5%)	1355 (26.3%)	3085 (25.1%)
Above 1 *SD*	795 (20.8%)	244 (19.7%)	551 (21.4%)

^a^ Due to the number of levels, data for county of residence is not shown.

^b^ Standardized as described in the Supplemental Material.

**FIGURE 1:**
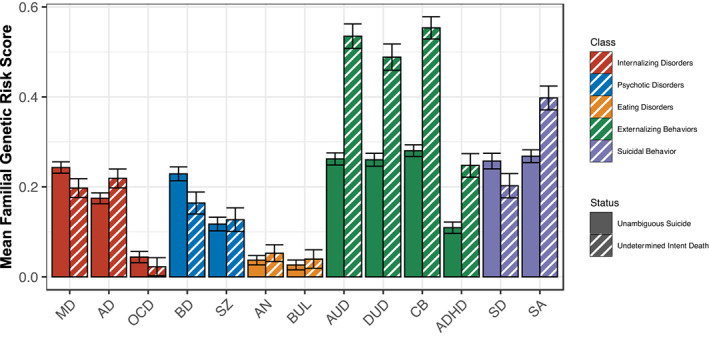
Mean (95% confidence intervals) family genetic risk scores for a range of behavioral outcomes, depicted for individuals whose deaths were unambiguous suicides (solid bars) versus of undetermined intent (striped bars). Color coding reflects thematically related registrations. AD, anxiety disorders; ADHD, attention deficit hyperactivity disorder; AN, anorexia nervosa; AUD, alcohol use disorder; BD, bipolar disorder; BUL, bulimia nervosa; CB, criminal behavior; DUD, drug use disorder; MD, major depression; OCD, obsessive compulsive disorder; SA, suicide attempt; SD, suicide death; SZ, schizophrenia

**FIGURE 2:**
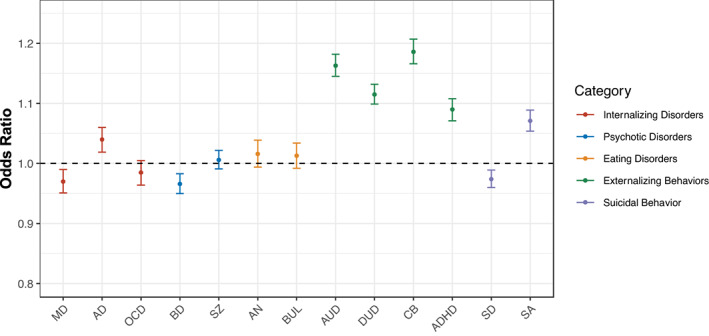
Odds ratios and 95% confidence intervals from univariate logistic regressions. Family genetic risk scores for a range of behavioral outcomes were tested for their association with suicide death status (death of undetermined intent versus unambiguous suicide), controlling for year of birth, sex, mean parental education, and municipality. Color coding reflects thematically related registrations. The dashed black line represents the null hypothesis (odds ratio = 1). An odds ratio >1 indicates higher odds of undetermined intent classification. AD, anxiety disorders; ADHD, attention deficit hyperactivity disorder; AN, anorexia nervosa; AUD, alcohol use disorder; BD, bipolar disorder; BUL, bulimia nervosa; CB, criminal behavior; DUD, drug use disorder; MD, major depression; OCD, obsessive compulsive disorder; SA, suicide attempt.SD, suicide death; SZ, schizophrenia

In preliminary comparisons (Figure 1), we observed higher FGRS among those with UDI deaths, relative to unambiguous suicide deaths, for the following outcomes: anxiety disorders, AUD, DUD, CB, ADHD, and SA. We observed lower FGRS for MD, BD, and suicide death among individuals with UDI deaths. These differences were further reflected in univariate logistic regressions that estimated the associations between each FGRS and death status (UDI death versus unambiguous suicide; Figure 2) while controlling for year of birth, sex, mean parental education, and county.

### Multivariate analyses

We next used LASSO to identify the most parsimonious multivariate model that minimized prediction error for the outcome variable (UDI versus unambiguous suicide). The selection process yielded a model that included 8 (of 12 potential) FGRS: those for MD, OCD, BD, AUD, DUD, CB, ADHD, and SD. Odds ratios are provided in Table 2, Model 1. Higher FGRS for MD, BD, and SD were associated with a decreased likelihood of UDI death (relative to unambiguous suicide) (ORs = 0.91–0.96), while higher FGRS for AUD, DUD, CB, and ADHD were associated with increased likelihood of UDI death (ORs = 1.04–1.12). While the inclusion of FGRS for OCD helped reduce prediction error, this variable was not itself associated with the outcome. The model including these 8 FGRS yielded a very minor improvement in the AUC relative to a model consisting only of covariates (AUC = 0.62 [0.61; 0.62] versus AUC = 0.59 [0.58; 0.59], respectively).

**TABLE 2:** Odds ratios (95% confidence intervals) from the LASSO regressions estimating the association between family genetic risk scores (FGRS) and suicide death status (unambiguous suicide versus death of undetermined intent).

	Model 1^a^		Model 2^b^	
	Odds ratio	95% CI	Odds ratio	95% CI
FGRS outcome				
Major depression	0.91	0.89; 0.93	0.92	0.90; 0.95
Obsessive compulsive disorder	0.98	0.96; 1.00	0.98	0.96; 1.00
Bipolar disorder	0.96	0.94; 0.97	0.98	0.96; 1.00
Alcohol use disorder	1.12	1.10; 1.14	1.03	1.01; 1.05
Drug use disorder	1.04	1.02; 1.06	1.01	0.99; 1.03
Criminal behavior	1.12	1.10; 1.15	1.03	1.01; 1.06
Attention deficit hyperactivity disorder	1.05	1.03; 1.07	1.03	1.01; 1.05
Suicide death	0.95	0.94; 0.97	0.96	0.94; 0.97
Phenotypic registration^c^				
Major depression	n/a	n/a	0.52	0.49; 0.55
Obsessive compulsive disorder	n/a	n/a	0.87	0.65; 1.16
Bipolar disorder	n/a	n/a	0.72	0.64; 0.81
Alcohol use disorder	n/a	n/a	5.12	4.85; 5.40
Drug use disorder	n/a	n/a	2.13	2.00; 2.27
Criminal behavior	n/a	n/a	1.30	1.22; 1.38
Attention deficit hyperactivity disorder	n/a	n/a	1.02	0.84; 1.23

*Note*: An odds ratio > 1 indicates higher odds of undetermined intent death.

^a^ Model 1 includes sociodemographic covariates (year of birth, sex, mean parental education, and municipality) and the eight selected FGRS.

^b^ Model 2 includes sociodemographic covariates, the eight selected FGRS, and the phenotypic registration status corresponding to each of the FGRS (except suicide death) from Model 1.

^c^ This refers to the registrations for the decedent. Because suicide death is one level of the outcome variable, it was not included as a predictor.

Based on the FGRS selected via LASSO, we conducted a subsequent analysis in which we included the status of phenotypic registrations corresponding to the selected FGRS (with the exception of suicide death). These findings are presented in Table 2, Model 2. In this model, odds ratios for FGRS were attenuated, but many remained significantly different than the null (i.e., 95% CIs did not overlap 1). FGRS for MD and suicide death were associated with a decreased likelihood of UDI death (ORs = 0.92–0.96); FGRS for AUD, CB, and ADHD were associated with an increased likelihood of UDI death (ORs = 1.01–1.03). Odds ratios for the phenotypic registrations were overall stronger than those for FGRS (Table 2, Model 2). For example, the odds ratio for FGRS for MD in this fully adjusted model was 0.92 (0.90; 0.95), while the odds ratio for MD phenotypic registration was 0.52 (0.49; 0.55).

## DISCUSSION

In the current study, we sought to provide genetic context to questions regarding whether the inclusion of deaths of undetermined intent is warranted in suicide research. We provide empirical evidence that genetic factors, which contribute substantially to the overall risk of suicidal behavior (Docherty et al., 2020; Edwards, Ohlsson, Mościcki, et al., 2021; Mullins et al., 2022), differ to a small but – in some cases – significant degree across unambiguous suicides and deaths of undetermined intent. In addition, despite widespread genetic correlations between suicidal behavior and many manifestations of psychopathology (Docherty et al., 2020; Mullins et al., 2022), aggregate genetic liability to only select psychiatric outcomes differed between UDI deaths and unambiguous suicide deaths. Our findings indicate that, from a genetic perspective, etiologic differences between these groups are not pronounced, but suggest that decedents who have a higher liability to externalizing behaviors, or lower liability to major depression and suicide death, may present challenges with respect to determining intentionality of death.

Even those adjusted FGRS estimates that differ significantly from the null hypothesis (i.e., OR = 1) are of small effect size, particularly relative to the effect sizes observed for several of the phenotypic registrations. Standard polygenic risk scores based on individual‐level molecular data, when derived from and applied to psychiatric outcomes, vary in their estimated effects, but it is not unusual to see ORs that are higher than those in the current report (Campos et al., 2021; Fanelli et al., 2022; Johnson et al., 2021). However, the nature of our research question – i.e., whether the aggregate genetic risk can distinguish between members of two groups who might overlap considerably – is somewhat more nuanced than, for example, efforts to test whether polygenic scores are associated with more clearly delineated groups such as those with versus without schizophrenia. We therefore interpret the small effect sizes as evidence of correspondingly minor, albeit in some cases statistically significant, genetic differences across groups. In contrast, effect sizes for several of the phenotypic registrations were more pronounced. This, too, warrants additional investigation in future studies: A recent report found that a slight majority of suicide decedents did not have a known history of mental illness (Stone et al., 2018). This underscores the possibility that preconceptions about suicide being inextricably linked to mental illness (Obegi, 2021) fail to fully acknowledge the heterogeneity of social, medical, and psychological processes contributing to suicide, a concern that potentially extends to decisions made regarding UDI classification.

Importantly, large‐scale molecular genetic studies of suicide death have recently become feasible (Docherty et al., 2020). Interestingly, the Docherty et al. (2020) report described the process of suicide identification in their Utah sample as having been made “quite conservatively” – presumably, excluding UDI deaths – and found that suicide decedents exhibited elevated polygenic scores for behavioral disinhibition, which is related to, but not interchangeable with, impulsivity (Joyner et al., 2021; Meda et al., 2009) and externalizing tendencies. They also observed higher polygenic scores for depression among suicide decedents.

While progress is being made with the genetics of suicidal behavior overall (Docherty et al., 2020; Levey et al., 2019; Mullins et al., 2022; Polimanti et al., 2021; Wendt et al., 2021) – i.e., relative to the overall population – we are unaware of other studies that have addressed potential genetic differences between unambiguous suicides and UDI deaths, and the current findings should be interpreted with caution pending replication. If molecular genetic data and UDI status is available within the same sample, that presents an opportunity to apply methodological advances to identify individual genetic variants or gene sets that might distinguish between UDI deaths and unambiguous suicides. For example, GenomicSEM (Grotzinger et al., 2019) could be used to identify variants with outcome‐specific effects. Given the current results, we might expect that such approaches would identify qualitative or quantitative differences across UDI deaths and unambiguous suicides for variants implicated in depression, substance use problems, and impulsivity. As noted above, impulsivity is a complex and multifaceted construct (Whiteside & Lynam, 2001), which is also reflected in its underlying genetic structure (Gustavson et al., 2020); this underscores the importance of refined phenotyping where possible.

Discrepancies in death classification as a function of mental health history may reflect medical examiners' hesitancy to classify a death as a suicide if the decedent did not have a known history of psychopathology. In contrast, a history of substance use disorder might lead medical examiners to be more likely to classify a death as UDI, creating a higher proportion of less reliable estimates for suicide in this group. Differential classifications could also reflect a bias that individuals with a history of psychoactive substance use or other impulsive behavior (e.g., ADHD, criminality) are less likely to have been acting intentionally. This possibility could be explored further by examining the intersection of UDI deaths, psychiatric/behavioral history (to include SUD and criminal behavior), and method of death: If decedents who died via the same method are less likely to be classified as unambiguous suicide deaths when they have a history of impulsive behavior, this may be considered evidence of classification bias.

It is not possible to know with certainty what proportion of UDI deaths represent true suicides, due to their very nature. As noted above, many studies have found that a nontrivial proportion of UDI deaths could be confidently reclassified as suicides; however, a parallel large‐scale effort would require careful follow‐up for which resources are limited. Importantly, our results do not suggest that all UDI decedents should be classified as suicide deaths: These findings merely represent an effort to clarify whether genetic factors provide clues to distinct etiologies, which may be informative in selecting an analytic approach in future studies.

Aside from including UDI deaths as suicides without further scrutiny, several analytic options exist: (i) exclusion of UDI deaths from suicide analyses altogether; (ii) inclusion of UDI deaths in the unaffected/control group; (iii) inclusion of UDI deaths as suicides in primary analyses, accompanied by a sensitivity analysis that excludes them; and (iv) treatment of UDI deaths and unambiguous suicide deaths separately altogether. Here, the research question should probably inform the selection decision. Findings from our fully adjusted model (Table 2, Model 2) suggest that any genetic differences between UDI deaths and unambiguous suicides are so minor that they might not be impactful from a genetic perspective, in which case option (iii) above would be a conservative alternative to the inclusion of UDI deaths as suicides without caveats. In contrast, the relatively strong associations between phenotypic registrations and suicide status (unambiguous versus UDI) might argue for option (iv) in analyses examining psychiatric comorbidity.

Our findings must be considered in the context of several limitations. First, our FGRS, while conceptually related to molecular polygenic scores, do not reflect measured genetic factors, and are dependent upon the presence of family members' registrations for phenotypes of interest (e.g., depression, ADHD, criminal behavior). Replication of the current findings using traditional polygenic scores is therefore necessary. Second, we were unable to account for individual‐level variation in judgment applied by coroners/medical examiners in their classification of deaths as suicides or UDI. We included county as a covariate in our analyses to address potential differences in local custom, but cannot exclude the possibility that some medical examiners are especially disinclined to classify a death in a particular way, which would contribute to further lack of clarity. Third, while our findings leverage nationwide data available in Sweden, they might not generalize to other countries or cultures, particularly in the context of differences in the prevalences of, conceptualizations of, or stigma around mental health and suicide.

## CONCLUSION

In summary, we found, using Swedish national medical and criminal registries, minor differences in genetic liability across individuals who died by suicide versus those whose deaths were classified as being of undetermined intent. Specifically, the latter exhibited higher genetic liability to substance use disorders, criminal behavior, and ADHD, and lower genetic liability to depression and suicide. In multivariate models controlling for registration for the corresponding phenotypes, the effect sizes of genetic risk were attenuated and, in some cases, no longer significant, indicating that genetic differences between groups are minimal. Our results indicate that these groups are etiologically similar from a genetic perspective, which has implications for the inclusion of UDI deaths in genetic analyses of suicide.

## AUTHOR CONTRIBUTIONS

Alexis C. Edwards: Conceptualization, Writing – Original Draft, Funding Acquisition, Investigation, Project Administration, Methodology. Henrik Ohlsson: Formal Analysis, Writing – Review and Editing, Data Curation, Methodology. Eve Mościcki: Writing – Review & Editing. Jan Sundquist: Resources, Writing – Review and Editing, Funding Acquisition. Casey Crump: Writing – Review and Editing, Funding Acquisition. Kenneth S. Kendler: Writing – Review and Editing, Funding Acquisition. Kristina Sundquist: Resources, Funding Acquisition, Writing – Review and Editing, Funding Acquisition.

## FUNDING INFORMATION

This study was supported by NIH grants MH129356, AA027522, AA023534, and DA030005; and by the Swedish Research Council, as well as ALF funding from Region Skåne.

## CONFLICT OF INTEREST

None.

## ETHICAL APPROVAL

We secured ethical approval for this study from the Regional Ethical Review Board in Lund, and no participant consent was required as the analyses were based on secondary data (No. 2008/409 and later amendments).

## Supporting information


Table S1.

## Data Availability

Data used in this study were obtained through Statistics Sweden and are not publicly available.
