# Supplementary material for: Genetic differences between suicide deaths and deaths of undetermined intent
Source: Suicide Life Threat Behav. 2022 Oct 31;53(1):100–9. doi: 10.1111/sltb.12926 (PMC9908835; doi:10.1111/sltb.12926)
Supplement: Supplementary file 1 — Table S1. [file SLTB-53-100-s001.docx]

**Supplemental Material**

**Supplemental Table 1.** Definition of phenotypes and variables.

|  | Registers Used | Definition |
| --- | --- | --- |
| Suicide death (SD) | The Swedish mortality register (1969-2018) | IC10: X60-X84 and Y10-Y34  For registrations prior to 1997  ICD9: E950-E959 and E980-E989  UDI : Y10-Y34 and E980-E989 |
| Suicide Attempt (SA) | The Swedish Hospital Discharge Register (coverage 1973-2018); Outpatient Care Register (national coverage 2001-2018); Primary Care Registry (Partly coverage from 1999-2018) | IC10: X60-X84 and Y10-Y34  For registrations prior to 1997  ICD9: E950-E959 and E980-E989 |
| Major Depression (MD) | The Swedish Hospital Discharge Register (coverage 1973-2018); Outpatient Care Register (national coverage 2001-2018); Primary Care Registry (Partly coverage from 1999-2018) | ICD-8: 296.2, 298.0, 300.4; ICD-9: 296.2, 296.4, 298.0, 300.4; ICD-10: F32, F33. |
| Anxiety Disorder (AD) | The Swedish Hospital Discharge Register (coverage 1973-2018); Outpatient Care Register (national coverage 2001-2018); Primary Care Registry (Partly coverage from 1999-2018) | ICD-8: 300.0, 300.2 ; ICD-9: 300A, 300C; ICD-10: F40, F41 |
| Obsessive-Compulsive Disorder (OCD) | The Swedish Hospital Discharge Register (coverage 1973-2018); Outpatient Care Register (national coverage 2001-2018); Primary Care Registry (Partly coverage from 1999-2018) | ICD-9: 300D; ICD-10: F42 |
| Bipolar Disorder (BD) | The Swedish Hospital Discharge Register (coverage 1973-2018); Outpatient Care Register (national coverage 2001-2018); Primary Care Registry (Partly coverage from 1999-2018) | ICD-8: 296.1, 296.3, 296.8, 296.9, 298.1; ICD-9: 296A, 296C, 296D, 296E, 296W, 298B; ICD-10: F30, F31 |
| Schizophrenia (SZ) | The Swedish Hospital Discharge Register (coverage 1973-2018); Outpatient Care Register (national coverage 2001-2018); Primary Care Registry (Partly coverage from 1999-2018) | ICD-8: 295.1, 295.2, 2953, 295.9, 295.6; ICD-9: 295B, 295C, 295D, 295G, 295X; ICD-10: F200, F201, F202, F203, F205, F209 |
| Bulimia (BUL) | The Swedish Hospital Discharge Register (coverage 1973-2018); Outpatient Care Register (national coverage 2001-2018); Primary Care Registry (Partly coverage from 1999-2018) | ICD-10: F502, F503 |
| Anorexia Nervosa (AN) | The Swedish Hospital Discharge Register (coverage 1973-2018); Outpatient Care Register (national coverage 2001-2018); Primary Care Registry (Partly coverage from 1999-2018) | ICD-9: 307B; ICD-10: F500 |
| Alcohol Use Disorder (AUD) | The Swedish Hospital Discharge Register (coverage 1973-2018); Outpatient Care Register (national coverage 2001-2018); Primary Care Registry (Partly coverage from 1999-2018); the Swedish Drug Register (2005-2018); the Swedish Mortality Register, and the Swedish Criminal Register (1973-2018) and the Swedish Suspicion Register (1998-2018) | Alcohol Use Disorder (AUD) was identified in the Swedish medical and mortality registries by ICD codes: ICD9: V79B, 305A, 357F, 571A-D, 425F, 535D, 291, 303, 980; ICD 10: E244, G312, G621, G721, I426, K292, K70, K852, K860, O354, T51, F10); in the Crime Register by codes 3005, 3201, which reflect crimes related to alcohol abuse; in the Suspicion Register by codes 0004, 0005 (Only those individuals with at least two alcohol-related crimes or suspicion of crimes from both Crime Register and Suspicion Register were included); in the Prescribed Drug Register by the drugs disulfiram (Anatomical Therapeutic Chemical (ATC) Classification System N07BB01), acamprosate (N07BB03), and naltrexone (N07BB04). |
| Drug Use Disorder (DUD) | The Swedish Hospital Discharge Register (coverage 1973-2018); Outpatient Care Register (national coverage 2001-2018); Primary Care Registry (Partly coverage from 1999-2018); the Swedish Drug Register (2005-2018); the Swedish Mortality Register, and the Swedish Criminal Register (1973-2018) and the Swedish Suspicion Register (1998-2018) | Drug abuse (DA) was identified in the Swedish medical and mortality registries by ICD codes (ICD8: Drug dependence (304); ICD9: Drug psychoses (292) and Drug dependence (304); ICD10: Mental and behavioral disorders due to psychoactive substance use (F10-F19), except those due to alcohol (F10) or tobacco (F18)); in the Suspicion Register by codes 3070, 5010, 5011, and 5012, that reflect crimes related to DA; and in the Crime Register by references to laws covering narcotics (law 1968:64, paragraph 1, point 6) and drug-related driving offences (law 1951:649, paragraph 4, subsection 2 and paragraph 4A, subsection 2). DA was identified in individuals (excluding those suffering from cancer) in the Prescribed Drug Register who had retrieved (in average) more than four defined daily doses a day for 12 months from either of Hypnotics and Sedatives (Anatomical Therapeutic Chemical (ATC) Classification System N05C and N05BA) or Opioids (ATC: N02A). |
| Criminal behavior (CB) | Swedish Crime register (1973-2018) | All convictions excluding convictions for minor crimes like traffic violations. |
| Attention deficit hyperactivity disorder (ADHD) | The Swedish Hospital Discharge Register (coverage 1973-2018); Outpatient Care Register (national coverage 2001-2018); Primary Care Registry (Partly coverage from 1999-2018) | ICD-9: 314; ICD-10: F90 |
| Parental Education | The longitudinal integration database for health insurance and labor market studies (LISA) from 1990-2018 and The Swedish Census from 1970. | Highest achieved education measured in 1-7 levels that in turn were translated into number of years of education and then standardized with mean 0 and SD 1 by gender and year of birth. In the registers the variable are as follows:  1 - Pre-high school (7 years)  2 - High School (9 years)  3 - 11 years of upper secondary school  4 - 12 years of upper secondary school  5 - 14 years of post-secondary education  6 - 18 years of post-secondary education  7 - PhD education (21 years)  We used the mean parental education in the analysis. |
| County of residence | The register of total population | The counties of Sweden (Swedish: Sveriges län) are the top-level geographic subdivisions of Sweden. Sweden is today divided into 21 counties. We included this as a categorical variable in the analysis to account for potential differences in local custom with respect to classification of suicide deaths. |

**Supplemental Table 2.** Family Genetic Risk Score derivation.

| The dataset for the calculations includes:  Column1 = Identification number of the proband (Born 1932-1995)  Column2 = Identification number of the relative (1st to 5th degree relatives)  Column3 = Proportion of shared additive genetic effects (0.03125 to 0.50) with the proband  Column4 = Year of Birth of relative  Column5 = Sex of relative  Column6 = Age at registration for trait  Column7 = Age at end of follow-up (2018-12-31 or age at death, or age at emigration whichever came first) |
| --- |
| **Step 1:** Using all unique relatives with a registration for the disorder, we non-parametrically estimated the distribution of *Age at first registration*. The empirical distribution is used to obtain weights for relatives without a registration for the disorder, in order to account for the proportion of the time-at-risk period they had completed at the end of follow-up. For example, for relatives at age x at end of follow-up, the weight corresponds to the proportion of relatives registered for the trait that had been registration at age x. For relatives born prior to 1958 we subtracted age at the end of follow-up with the following formula: 1958 - Year of birth of relative. This modification was done to control for registration effects (i.e., most registers in Sweden start in 1973 suggesting that relatives from early birth cohorts do not have the possibility to be registered at younger ages). Note that all relatives with the disorder are weighted one. |
| **Step 2:** Transform the binary variable (trait yes/no) into a z-score based on the threshold for each trait. The underlying liability of the individual is not assessable. Instead, we estimated the mean of the underlying liability to obtain sex and birth decade specific Z-scores for relatives with the trait registration and relatives without the trait. We generate n random numbers from a N (0, 1) distribution and estimate the mean for relatives registered with the disorder (i.e., mean of the observations above the threshold) and for relatives without a registration (i.e., mean of all observation below the threshold). The thresholds are calculated for each decade of birth and sex. |
| **Step 3**: Correct for cohabitation effects. To estimate the cohabitation effect (i.e., “shared environment”), we created a database with all individuals in the Swedish population born in Sweden 1955-1990. We also included the number of years, during ages 0-15, that individuals resided in the same household as their biological father. We thereby were able to define two kinds of families: i) “not-lived-with” father families (offspring never resided for more than 1 year in the same household or in the same community as their biological father); ii) “lived-with” father (offspring resided a minimum of 13 year in the same household as their biological father. We performed a logistic regression model with the binary trait in offspring as outcome and the binary trait in father, type of father, and their interaction as predictors. We used the interaction term as the difference of effect between genes only and genes + environment. The same approach was performed for half-siblings where we compared those who were reared together versus reared apart. The following interaction terms were used in the calculations for each of our 7 main disorders:   \|  \| Parent/Children \| Siblings \| \| --- \| --- \| --- \| \| MD \| 0.80 \| 0.85 \| \| AD \| 0.87 \| 0.81 \| \| OCD \| 0.79 \| 0.74 \| \| BD \| 0.67 \| 0.77 \| \| SZ \| 0.93 \| 0.84 \| \| BUL \| * \| 0.87 \| \| AN \| * \| 0.88 \| \| AUD \| 0.99 \| 0.69 \| \| DA \| 0.92 \| 0.52 \| \| ADHD \| 0.42 \| 0.81 \| \| SA \| .66 \| .76 \| \| SD \| .70 \| .98 \| \| CB \| 0.87 \| 0.81 \| \|  \|  \|  \| \| *No reliable estimate due to very low prevalence rates in males – we therefore used the mean among all other traits \| \| \| |
| **Step 4:** Calculate the product for each relative using the four components:   1. Z-score (reflecting sex and year of birth adjusted rates) 2. Weight (reflecting the proportion of risk period they had completed) 3. Cohabitation effects 4. Proportion of shared genetic effects (0.03125 – 0.5) with the proband |
| **Step 5:** Average the product calculated in step 4 across all relatives to a proband. |
| **Step 6**: Correct for the number of relatives. We multiplied the results from step 5 with a shrinkage factor. Shrinkage factor (SF): B/(B+A/C). It produces more shrinkage if B and C are small and A is large.   1. the variance of the z-score of the disorder across all relatives, 2. the variance in the mean z-score across all probands, 3. the weighted number of relatives for each proband (sum of Column 3 across each proband). |
| **Step 7:** Correct for difference by year of birth and county differences. There are 21 counties in Sweden. For each proband we used the county they had resided in during the maximum number of years (measured from 1969 and onwards) We standardized (based on the total population) the risk score by year of birth and county of the proband into a z-score with mean 0 and SD 1. This was then used as the FGRS in the analyses. |

**Supplemental Table 3.** Association between the 12 proband-level phenotypic registrations, including psychopathology, substance use disorders, and criminal behavior, and UDI (controlling for Year of Birth, Sex, Parental Education, and County of Residence). Odds ratios (95% CIs) are provided.

|  | Odds Ratio (95% CI) |
| --- | --- |
| MD | 0.68 (0.64; 0.72) |
| AD | 1.12 (0.95; 1.33) |
| OCD | 0.91 (0.70; 1.19) |
| BD | 0.68 (0.61; 0.75) |
| SZ | 0.94 (0.84; 1.06) |
| AN | 0.65 80.41; 1.04) |
| BUL | 0.42 (0.20; 0.89) |
| AUD | 6.18 (5.88; 6.49) |
| DA | 3.50 (3.31; 3.70) |
| CB | 2.71 (2.58; 2.85) |
| ADHD | 2.07 (1.75; 2.46) |
| SA | 1.07 (1.01; 1.12) |
